# Uncovering a previously unknown function of polyphosphate in polyadenylated RNA‐induced amyloidogenesis of Hfq

**DOI:** 10.1111/febs.70445

**Published:** 2026-02-09

**Authors:** Kevin Mosca, Florian Turbant, Sambhasan Banerjee, Frank Wien, Richard R. Sinden, Véronique Arluison

**Affiliations:** ^1^ Laboratoire Léon Brillouin, UMR 12 CEA/CNRS Gif‐sur‐Yvette France; ^2^ Synchrotron SOLEIL Gif‐sur‐Yvette France; ^3^ Sanofi, Biochemistry and Biophysics mRNA Unit, mRNA Center of Excellence, Analytical Sciences Marcy l'Etoile France; ^4^ Université Grenoble Alpes, CNRS, LiPhy France; ^5^ CSSB Centre for Structural Systems Biology Deutsches Elektronen‐Synchrotron DESY Hamburg Germany; ^6^ Department of Chemistry, Biology and Health Sciences South Dakota School of Mines and Technology Rapid City SD USA; ^7^ Université Paris Cité, UFR SDV France

**Keywords:** bacterial amyloids, DNA and RNA compaction, Hfqliquid liquid phase separation (LLPS), nucleoid, polyadenylated RNA

## Abstract

Polyphosphate (polyP), a ubiquitous and highly conserved biopolymer, has emerged as a potential modulator of bacterial amyloidogenesis. In bacteria, polyP contributes to the formation of dense intracellular regions associated with transcriptional silencing. Gene regulation and chromatin organization are primarily controlled by nucleoid‐associated proteins (NAPs), including the highly conserved RNA chaperone Hfq. Recent studies suggest that polyP alters Hfq function, promoting genomic instability through increased mutagenesis and DNA damage. *In vitro*, Hfq interacts with polyP and nucleic acids to form phase‐separated condensates, a process mediated by its intrinsically disordered C‐terminal region (CTR). In this study, we investigated the impact of polyP on the amyloidogenic behavior of Hfq. Our results reveal that, contrary to expectations, polyP alone does not induce amyloid formation in the isolated CTR. However, in the presence of polyadenylated RNA, polyP significantly enhances Hfq amyloidogenesis. These findings suggest a previously unrecognized role for polyP in RNA‐mediated phase separation using amyloid self‐assembly and provide new insights into the molecular mechanisms underlying bacterial stress tolerance.

## Graphical abstract

Polyphosphate interaction with Hfq and RNA drives phase separation and amyloid self‐assembly, uncovering a previously unrecognized role for polyP in bacterial stress responses. Our findings reveal new insights into the molecular mechanisms of RNA‐mediated phase separation, highlighting the role of polyP as a key modulator of this process.

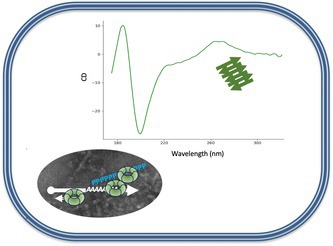

**Abbreviations**
bp: base pair
BR‐bodies: bacterial ribonucleoprotein bodies
CTR/NTR: C/N‐terminal region
HP‐bodies: Hfq‐PolyP bodies
K_D_
: equilibrium dissociation constant
k_on_
: association rate constant
LLPS: liquid liquid phase separation
NA: nucleic acid
PAP: poly(A) polymerase
polyA: polyadenylate
polyP: polyphosphate
PPK: polyphosphate kinase
SRCD: synchrotron radiation circular dichroism
sRNA: small noncoding RNA

## Introduction

Polyphosphate (polyP) is an ancient molecule that predates life. Formed by the dehydration of phosphate, it can be found in deep‐ocean steam vents [1, 2]. As a molecular precursor compatible with life, it has found its way into virtually every living organism and has a myriad of functions and molecular interactions [3]. Polymers of 100–1000 units provide a way to store energy in the high‐energy phosphoanhydride bond, as also occurs in ATP. The anionic polymer also has the capacity to chelate cationic metal ions (e.g., Ca^2+^ and Mg^2+^) and provide a surface for protein interaction or organization into membranes. In fact, polyphosphate can participate in the formation of membrane channels for Ca^2+^, PO_4_^−^, or DNA transport [4, 5].

From analysis of bacteria deficient in enzymes that produce polyP (polyP kinase, PPK) or organisms in which polyP is overproduced, it is evident that polyP is involved in a multitude of biological activities [3]. These include survival in stationary phase, response to metabolic stresses, sporulation, growth, motility, biofilm formation, virulence, and predation [2]. These effects are mediated by changes in the regulation of genes and operons involved in polyP metabolism and changes in levels of certain mRNAs. An interesting observation is that, in the absence of polyP, the nucleoid of bacteria can become compressed [6]. This suggests that the interactions of structural and regulatory proteins with the chromosome may be significantly altered leading to pleiotropic effects observed in PPK deficient mutants.

Proteins involved in bacterial nucleic acid shaping include Hfq. This bacterial RNA chaperone is a central regulator of RNA metabolism, playing a multifaceted role in gene expression control [7]. Structurally, Hfq forms a conserved hexameric torus (referred to as Sm core [8]), composed of its N‐terminal region (NTR), with six flexible C‐terminal regions (CTR) extending outward [7, 9]. The NTR is primarily responsible for RNA binding and annealing, facilitating interactions between small regulatory RNAs (sRNAs) and their mRNA targets [8, 10]. Although sRNAs modulate gene expression post‐transcriptionally in bacteria by affecting mRNA stability and translation, Hfq's influence extends beyond RNA pairing [11]. Notably, Hfq influences mRNA degradation independently of sRNAs [12, 13]. In Gram‐negative bacteria, polyadenylation by poly(A) polymerase (PAP) typically tags RNAs for exonucleolytic decay, and Hfq enhances PAP processivity [14]. On the other hand, Hfq protects RNA such as the *rpsO‐polyA* mRNA from RNase degradation [12]. Hfq indeed protects both the poly(A) tail and the *rpsO* internal binding site from ribonucleases degradation. The binding affinity of Hfq for polyadenylated *rpsO* RNA is exceptionally high, highlighting its role in RNA turnover and quality control [15].

While the NTR's role in RNA binding is well characterized [16], the function of the CTR remained poorly understood until recent studies uncovered its propensity to form amyloid‐like structures [17, 18]. Precisely, Hfq forms a functional amyloid. A functional amyloid serves beneficial physiological roles and its formation is often reversible or regulated. Pathological amyloids are misfolded aggregates associated with disease, and formation is typically irreversible [19]. The amyloidogenic CTR does not appear essential for most sRNA‐mediated regulation but contributes to specific RNA interactions. Furthermore, it is critical for Hfq's interaction with bacterial membranes and for DNA shaping [20, 21, 22]. Recent studies reveal the structural basis of Hfq's C‐terminal extension in DNA compaction, identifying an amyloid module that uniquely bridges and compacts multiple DNA molecules [22, 23]. These findings expand the known roles of amyloids to include genome structuring and suggest that conserved amyloid‐DNA interactions provide a fundamental mechanism influencing gene expression regulation in prokaryotes.

Building on these observations, the present study investigates how polyP influences the shaping of nucleic acids and Hfq self‐assembly. Understanding this novel function of polyP at the RNA interface could shed light on new bacterial regulatory mechanisms and may suggest strategies for future antimicrobial therapy.

## Results

### SRCD spectra of Hfq‐CTR in interaction with RNA

**Fig. 1:**
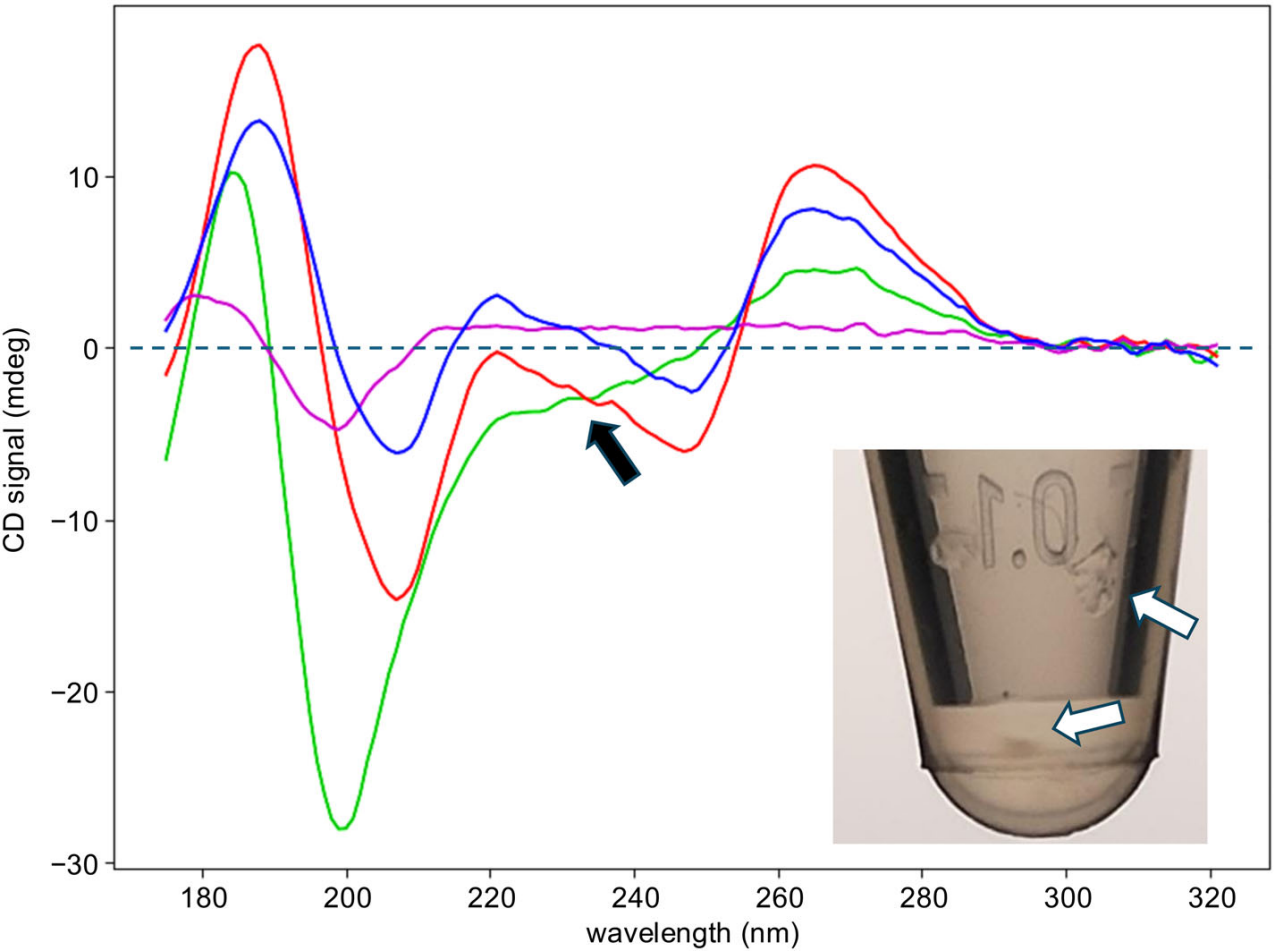
SRCD spectrum of CTR‐bound *rpsO‐polyA* RNA in the presence of polyP (green) with controls: *rpsO‐polyA* alone (blue), *rpsO‐polyA* + CTR in the absence of polyP (purple), and *rpsO‐polyA* in the presence of polyP without CTR (red). All SRCD spectra were acquired in triplicate. Note that polyP does not absorb UV light and does not present a CD spectrum. RNA/polyP/CTR ratio was 1 : 1 : 1 (expressed in nucleotides, phosphate, and peptide concentrations, respectively). The amyloidogenic nature of the CTR in the presence of polyP and RNA is indicated by the electronic transition observed at ~ 225 nm (black arrow). Samples were analyzed after at least 4 weeks to allow peptide self‐assembly. Note that studying the kinetics of Hfq‐CTR amyloid formation in the presence of polyP and RNA would be informative to assess its proposed role as an ‘accelerator’. Nevertheless, real‐time kinetic measurements were not feasible due to the long kinetics, and our conditions were based on previous optimization showing that *in vitro* self‐assembly of the CTR peptide requires approximately 4 weeks. Inset: the presence of phase‐separated condensates (white arrows) is indicative of a solid amyloid state, though it does not fully reflect the complexity of the phase transition.

**Fig. 2:**
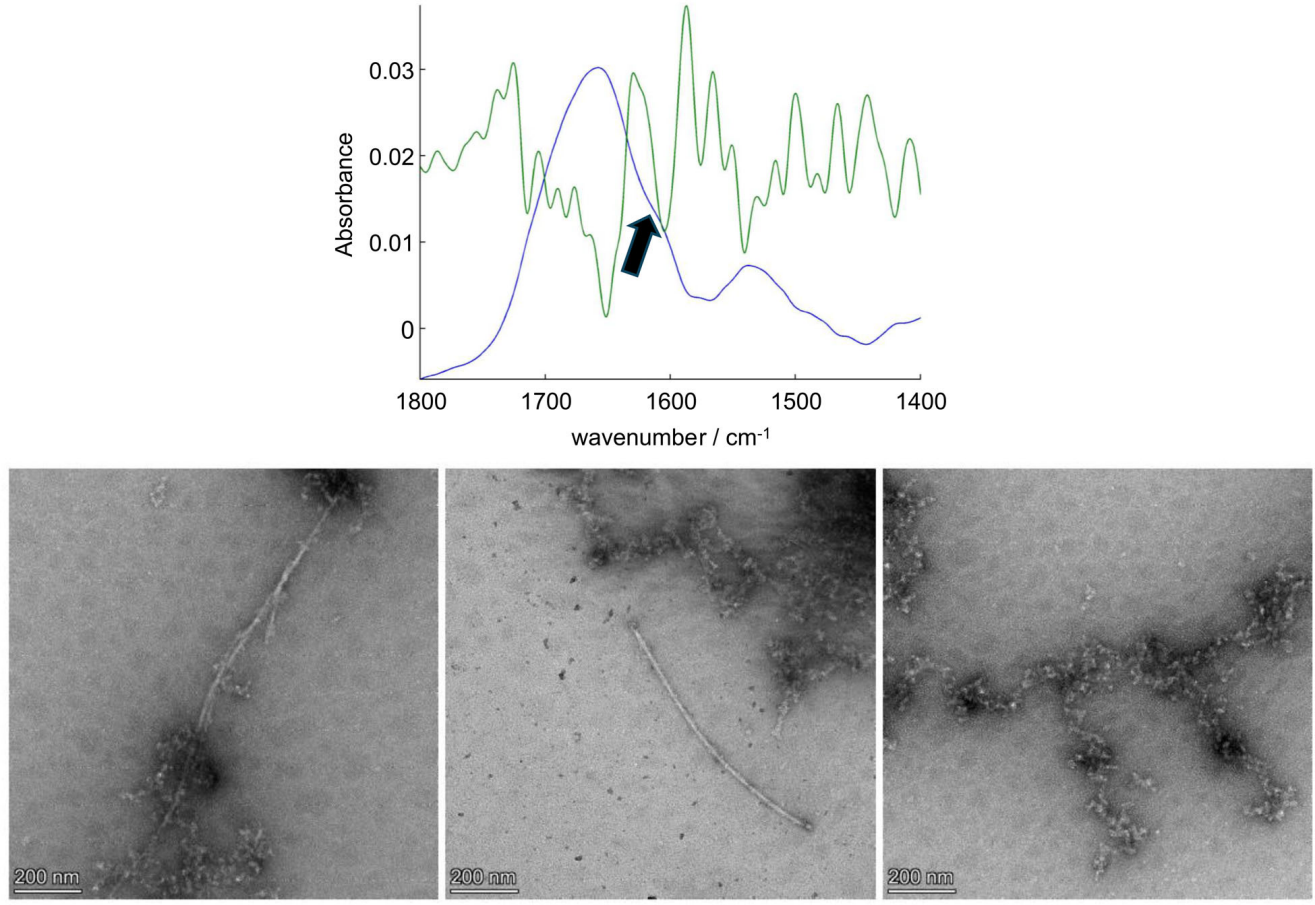
Top: FTIR spectrum of CTR in the presence of polyP and RNA. The blue trace shows the FTIR spectrum. The second‐derivative spectrum (green) reveals a band near 1605–1610 cm^−1^, a region typically associated with amyloid β‐sheet vibrations (black arrow) [18], although contributions from RNA base vibrations cannot be excluded [69, 70]. The presence of an amyloid signature was confirmed using FTIR spectroscopy [27]; Bottom: TEM imaging of Hfq fibers in the presence of polyP and *rpsO‐polyA*. Three examples are shown for statistical purposes. Note that the ligands induce amorphous and possibly nonamyloid aggregates, thereby obscuring the visualization of the amyloid architecture. The magnification was ×45 000. Scale bar 200 nm.

The SRCD spectra of Hfq were analyzed to investigate how polyP affects the structure of Hfq in the presence of RNA (Fig. 1). In earlier studies, we demonstrated that the CTR of Hfq forms amyloid‐like structures using a combination of complementary experimental approaches, including Thioflavin T (ThT) staining, transmission electron microscopy (TEM), gel shift assay, and Fourier transform infrared (FTIR) spectroscopy [18, 21, 24]. In the present study, synchrotron radiation circular dichroism (SRCD) spectroscopy was selected to characterize amyloid formation because it enables quantitative and dynamic monitoring of conformational transitions in solution. Several factors made other methods less adapted to our experimental conditions: ThT fluorescence is unreliable in the presence of nucleic acids, as the dye binds nonspecifically to nucleic acids, leading to false‐positive signals [25]; FTIR spectroscopy analysis is also complicated due to the strong vibrational bands of polyP and nucleic acids [26]; we can however confirm the presence of an amyloid signature using FTIR (Fig. 2) [27]; finally, in the absence of a high‐resolution reconstruction, TEM offers only morphological information and cannot resolve secondary structure features, and the presence of polyP or RNA could induce amorphous or nonamyloid aggregates [28]. Despite these limitations, we were able to confirm the presence of amyloid fibers using both FTIR and TEM (Fig. 2).

**Fig. 3:**
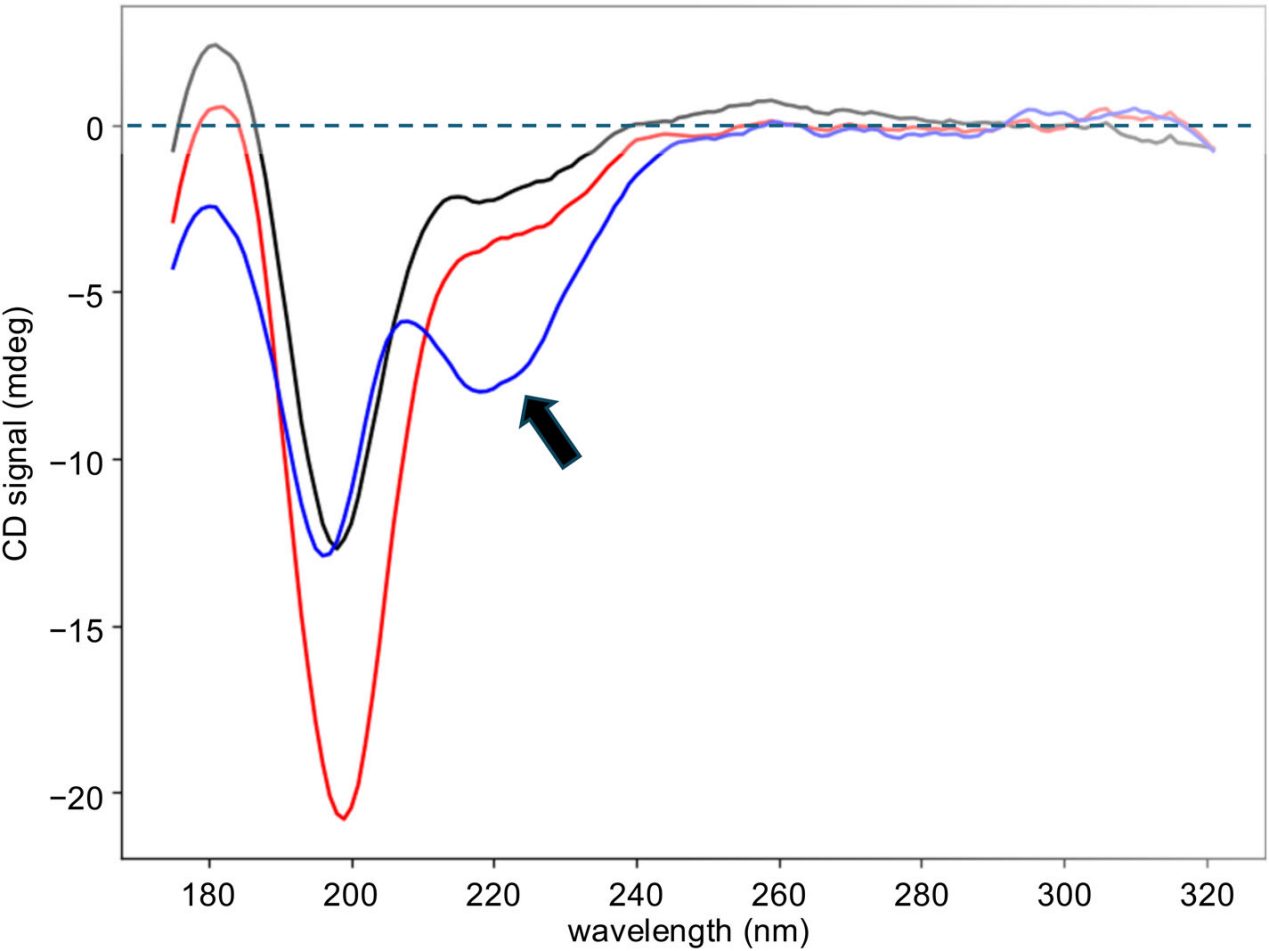
SRCD spectra of Hfq‐CTR in the presence of polyP: CTR alone (black); CTR + polyP (red) at a ratio 1 : 4 (expressed in peptide and phosphate concentrations, respectively; here, the amount of polyP was increased to promote amyloid formation); polymerized CTR after 5 months of incubation (blue, see the electronic transition observed at ~ 225 nm indicated by the black arrow).

As SRCD spectroscopy operates directly in aqueous solution to monitor structural changes and is minimally affected by the optical properties of polyP or nucleic acids, it was selected for our analysis. To focus specifically on the amyloid behavior of Hfq, we analyzed its isolated CTR. Note that while full‐length Hfq adopts an amyloid structure [29, 30, 31], the presence of its β‐rich Sm‐core complicates the interpretation of the results: the Sm ring in the NTR part, is mainly composed of β‐sheets but also of 10% of α‐helices, that partially mask the amyloid signature of the CTR [32]. Therefore, we focused on the 38‐amino acid (aa) CTR, which is capable of binding RNA and DNA to better understand the effect of polyP on Hfq amyloidogenesis [18, 21, 24, 31, 33]. For this analysis, we used the 3′ end of *rpsO‐polyA* RNA as a model, as Hfq has the highest affinity for such A‐rich sequences (*rpsO‐polyA*:Hfq equilibrium dissociation constant K_D_ ~ 100 pm) [12]. The SRCD spectra of Hfq‐CTR in the presence of *rpsO‐polyA* RNA and/or polyP are shown in Fig. 1. Spectra were recorded with an RNA/polyP/CTR ratio of 1 : 1 : 1 (expressed in nucleotides, phosphate, and peptide concentrations, respectively). Notable spectral changes in the 220 nm region are indicative of amyloid formation [21, 24] (see also Fig. 3), while changes in the 260–280 nm region correspond to changes in base tilting or base pairing in the RNA [34, 35]. Specifically, we observed that the addition of polyP to CTR in the absence of RNA did not result in significant changes to the SRCD spectrum shape (Fig. 3). In contrast, in the presence of the polyadenylated RNA, polyP induced amyloidogenesis of CTR (see Fig. 1, black arrow), while *rpsO‐polyA* alone did not exhibit such effect within the same timescale. This amyloid structure can be confirmed by the observation of condensates formed *in vitro* (see Fig. 1 inset, white arrows). Interestingly, we also observed that polyP affected the RNA spectrum and seemed to stabilize the RNA structure, as indicated by the increased intensity, at least in the base pairing region (260–280 nm) where the protein does not absorb. The effect of polyP on the helical structure of the RNA (observed in the region 180–200 nm) is more complex to analyze, as both the protein and RNA absorb in this region and undergo structural and spectral changes.

Recent studies supported a model in which polyP may exert its effects by inducing a conformational change in proteins [36, 37]. As shown on Fig. 3, if polyP does not induce amyloidogenicity in the absence of RNA, it seems to increase β‐sheet content. The secondary structure compositions were analyzed using SRCD spectra and Bestsel (https://bestsel.elte.hu). Consistent with prior findings, the peptide adopts β‐sheet configurations (~ 36%) [38]. When the peptide interacts with PolyP, the β‐sheet content increased to ~ 41%.

### Impact of the polyA tail on Hfq self‐assembly

**Fig. 4:**
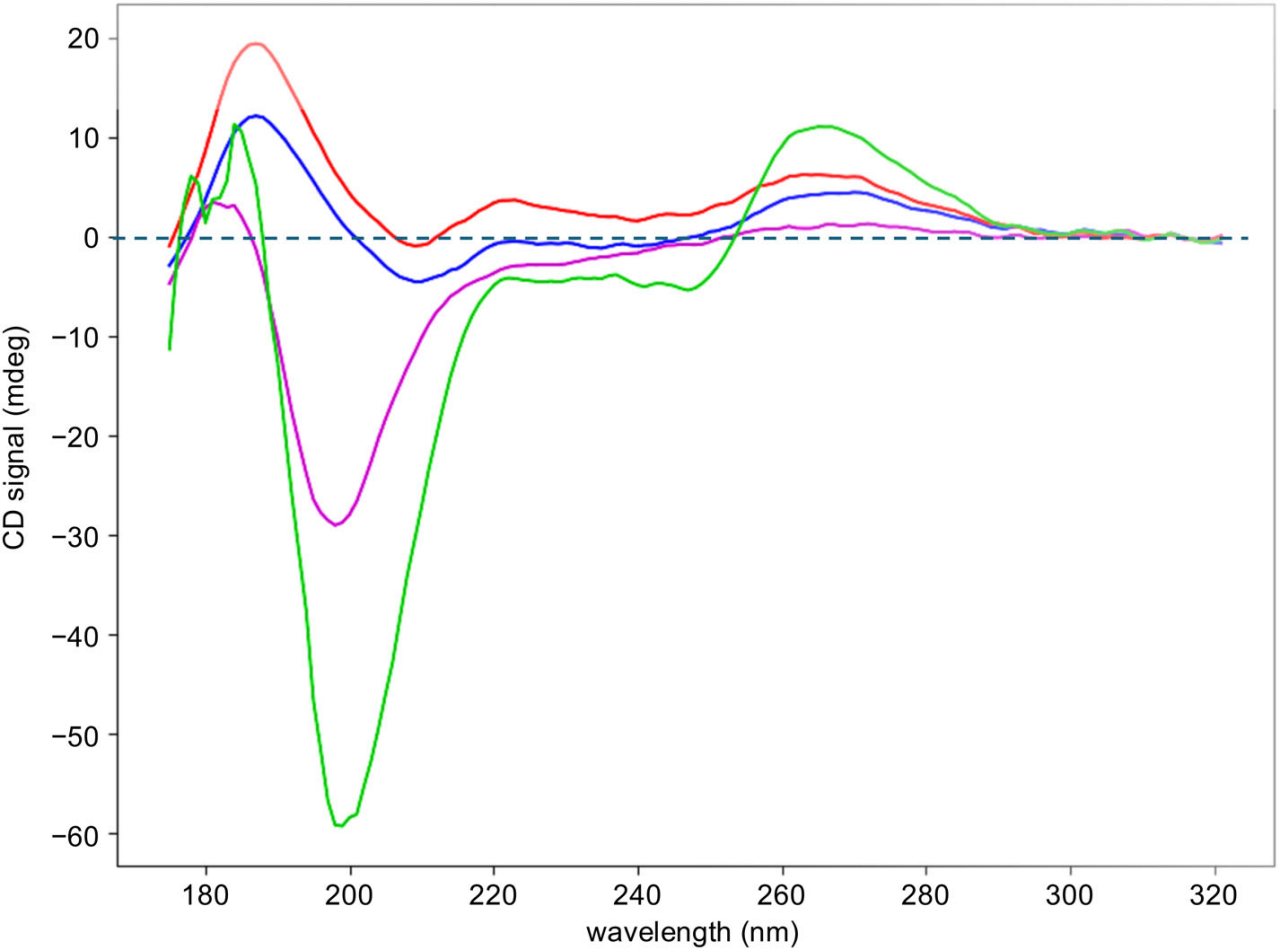
SRCD spectrum of CTR‐bound *rpsO* RNA in the presence of polyP (green) with controls: *rpsO* alone (blue), *rpsO* + CTR in the absence of polyP (purple), and *rpsO‐polyA* in the presence of polyP without CTR (red). The ratio used was the same as that in Fig. 1. In this case, the electronic transition at ~ 225 nm is not observed.

Recent studies have suggested a specific role of polyP in scaffolding Hfq within condensates, selectively stabilizing polyadenylated RNAs [39]. We thus analyzed the impact of the absence of the polyA tail on Hfq amyloidogenicity. A distinct behavior (absence of red shift) in the 200–220 nm region showed that nonpolyadenylated RNA did not induce the formation of the amyloid structure (Fig. 4), in contrast to *rpsO‐polyA* (Fig. 3). Meanwhile, alterations in the 260–280 nm range persist, suggesting that changes in RNA base tilting or pairing also occurred in the absence of the polyA tail.

### SRCD spectra of Hfq‐CTR in interaction with DNA

**Fig. 5:**
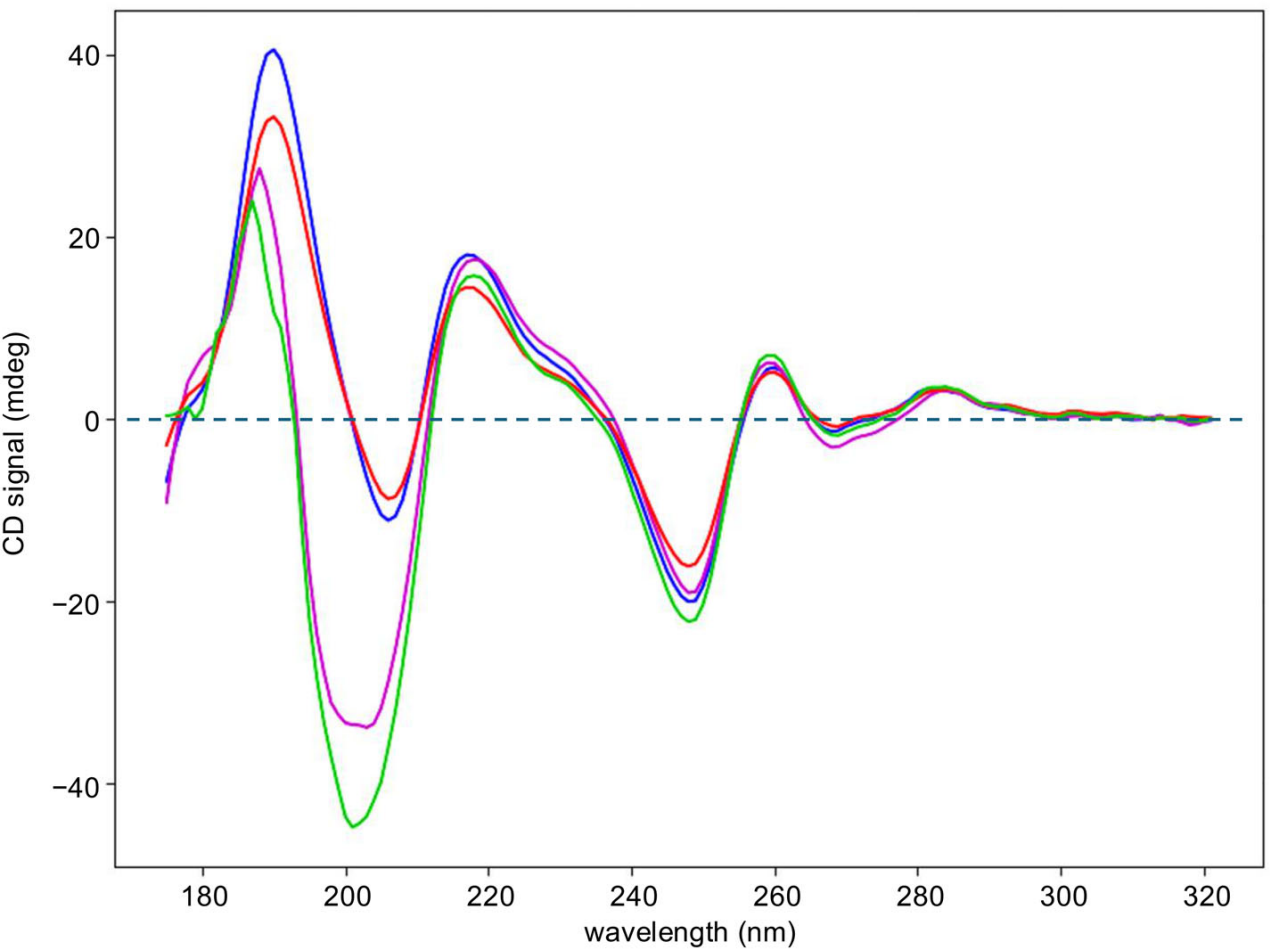
SRCD spectrum of CTR‐bound DNA in the presence of polyP (green) with controls: DNA alone (blue), DNA + CTR in the absence of polyP (purple), and DNA in the presence of polyP without CTR (red). DNA/polyP/CTR ratio was 4 : 1 : 1 (expressed in bp, phosphate, and peptide concentrations, respectively). The electronic transition at ~ 225 nm is not observed.

Finally, we analyzed the impact of DNA on CTR amyloidogenicity. PolyP has also been shown to form a three‐component condensate with AT‐rich DNA and the RNA chaperone protein Hfq suggesting a role for PolyP and Hfq in the formation of bacterial chromatin [40]. The DNA model selected for our analysis was a ~ 60‐bp homo‐polymer (dA : dT), as Hfq has the highest affinity for A‐rich sequences both *in vitro* and *in vivo* [41, 42]. This model was used previously in different studies [21, 24, 43]. As shown in Fig. 5, in the presence of DNA and polyP, the CTR did not self‐assemble more efficiently than without A‐rich DNA sequence, in contrast to the result with polyadenylated RNA.

## Discussion

In this work, we conducted a series of experiments to observe the structural changes induced by polyP on Hfq‐CTR, both alone and in the presence of DNA or RNA. Contrary to expectations [44], we found that polyP alone does not induce CTR amyloidogenesis. However, we observed that polyP promotes Hfq amyloidogenicity in the presence of a polyadenylated RNA. This effect appears to be specific and cannot be confirmed when DNA or the same RNA in its nonpolyadenylated form are used within the same experimental timeframe. Nevertheless, slower kinetics of amyloid formation may occur, which are not captured under our experimental conditions. Also note that although the *in vitro* conditions used are slow compared to physiological stress responses, *in vivo* conditions, including the presence of Hfq‐NTR, molecular crowding and other potential cofactors, could substantially accelerate amyloid formation. PolyP is therefore considered as a likely accelerator *in vivo*, although direct evidence remains to be established. Note that our observation contrasts with previous studies involving α‐Synuclein, the pathological hallmark of Parkinson's disease, and CsgA, the major component of curli fibers in biofilm [44, 45]. In both cases, polyP acts as an effective nucleation compound for amyloid formation and significantly enhances amyloidogenesis in the absence of nucleic acids [45].

Previous studies have supported a model in which polyP may influence protein conformation, potentially inducing structural changes [36, 37]. Hfq‐CTR SRCD spectra support this hypothesis, showing an increase in β‐sheet content in the presence of polyP. In contrast to CsgA and α‐Synuclein, Hfq amyloidogenicity is however not affected by polyP in the absence of RNA (Fig. 1). Importantly, CTR amyloidogenicity takes several days to develop *in vitro*, even with the presence of nucleic acid accelerators [24]. Therefore, polyP may also accelerate this process by promoting amyloid scaffold and phase separation *in vivo*. The mechanism underlying polyP's effect on CTR amyloid assembly remains unknown, and requires further investigation. Two nonmutually exclusive mechanisms may contribute. First, polyP may act as a bridge between the positively charged regions of Hfq and negatively charged RNA, stabilizing ternary complexes and promoting nucleation. Second, polyP may influence the phase behavior of Hfq‐RNA condensates, facilitating their maturation from dynamic liquid droplets into solid amyloid structures.

**Fig. 6:**
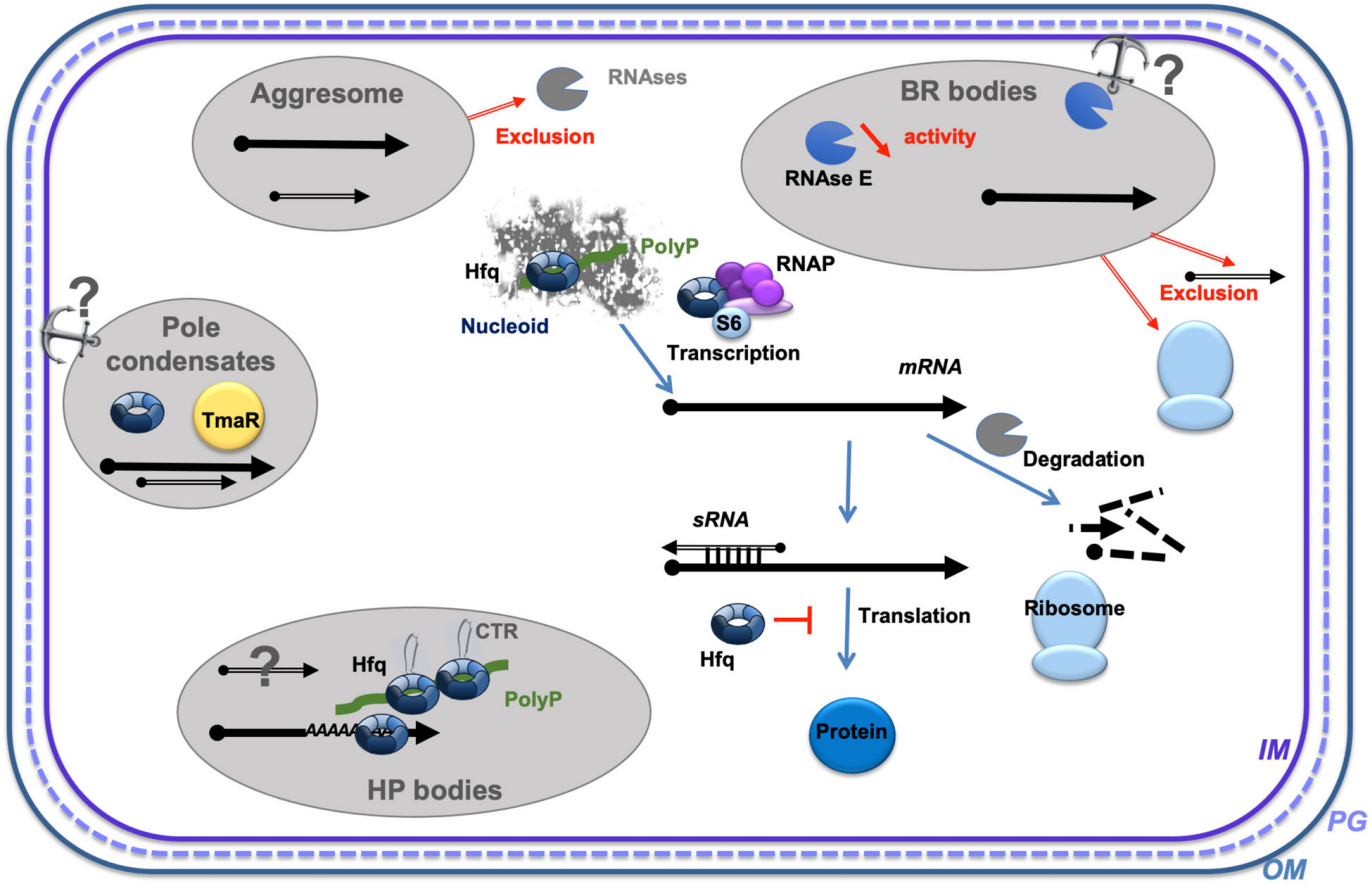
Bacterial membrane‐less RNA‐protein assemblies include HP‐bodies, BR‐bodies, aggresomes, and pole condensates. They all play crucial roles in organizing and regulating RNA transactions, in particular under stress conditions [56, 71]. (a) HP‐bodies (Hfq‐PolyP bodies) are centered around the RNA chaperone Hfq, its CTR and polyP, and act as hubs for polyadenylated mRNA‐mediated gene regulation [39]. Taking into account the function of the RNA chaperone in facilitating sRNA:mRNA pairing to modulate translation and degradation, the presence of sRNA in HP‐bodies is also likely, even if not yet proven. (b) BR‐bodies (Bacterial Ribonucleoprotein bodies) emerge under stress conditions, sequestering RNAs and proteins such as the major endonuclease RNAse E to protect cellular resources [47, 72]. They regulate RNA metabolism by switching between liquid‐like states that promote mRNA decay during growth and more rigid, solid‐like states that store mRNA under stress [47]. (c) Aggresomes, primarily known for sequestering misfolded proteins [73], can indirectly influence RNA metabolism by altering the availability of RNA‐binding proteins or ribosomes, especially during stress [54]. (d) Finally, pole condensates spatially organize RNAs at the cell poles [46]. They contain both Hfq and the pole‐localizer TmaR. Together, these condensates enable bacteria to dynamically compartmentalize RNA processing, storage, and quality control, allowing rapid adaptation to environmental changes. In parallel, in the nucleoid, polyP and Hfq also form condensates with DNA, impacting bacterial chromatin structure [40]. Hfq may also self‐assemble with transcription (RNA polymerase, RNAP) and translation factors (S6) [74]. sRNA regulators controlling mRNAs are depicted as open arrows; mRNAs as thick black lines; 5′ and 3′ of RNAs are depicted by a ‘ball and arrow head’, respectively; Hfq is depicted as a blue toroidal hexamer, phase‐separated bodies as gray ellipses, polyP as a green line; gray anchors symbolize a possible interaction with cellular membrane. Hfq‐dependent negative regulations are indicated by red T‐shape lines; the dotted line symbolizes the peptidoglycan (PG) between the outer and inner membranes (OM/IM).

Hfq and RNAs can phase separate in cells [46, 47], and recent analyses show that stresses influence this process. For instance, *E. coli* cells subjected to nitrogen starvation synthesize polyP, which facilitates the assembly of Hfq into phase‐separated complexes [39]. These structures were referred initially as BR‐bodies (bacterial ribonucleoprotein bodies) [47, 48] or more specifically as HP‐bodies (Hfq‐PolyP bodies) when Hfq is present [39]. Although HP‐body is not a formally established term, for the sake of simplicity and to facilitate comparison with other studies, we will refer to these structures as HP‐bodies. These bacterial bodies are believed to play a role in bacterial adaptation to extreme environmental conditions and stress [39, 47]. Note that Hfq condensates can also assemble in the absence of polyP, which is not essential for their formation (Fig. 6).

Traditionally, mRNA degradation has been considered as a cytosolic process controlled by diffusible RNA and proteins. The discovery that mRNAs can be spatially sequestered within phase‐separated compartments, where transcript stability and availability are influenced by physical segregation, adds a new dimension to the control of mRNA stability. While Hfq amyloid formation may protect mRNAs during stress, stable or irreversible aggregation could compromise Hfq's RNA chaperone activity. The physiological impact of this assembly depends critically on its stability and, importantly, its capacity for reversibility, which would allow Hfq to regain its regulatory function once stress conditions subside. We previously observed that Hfq amyloid self‐assembly is reversible [31, 49], consistent with the behavior of functional amyloids [50, 51]. Indeed, many functional amyloid assemblies have evolved mechanisms enabling their controlled reversal, allowing them to disassemble in response to specific cellular cues when their fibrillar state is no longer required. This suggests that disassembly of the amyloid state may contribute to post‐stress recovery [51]. Recent studies have shown that under stress, BR‐bodies become storage reservoirs [47]. This transition is driven by ribosomal depletion, but intriguingly, these condensates also appear to sequester RNAse E and RNA degradosome components [52, 53]. One possibility is that the BR‐body solid‐like state reduces diffusion and the k_on_ rate constant, which describes the rate at which the RNAse and RNA substrate bind to form an enzyme–substrate complex, slowing enzymatic activity [53]. Note that these BR‐bodies differ from aggresomes, where condensates exclude ribonucleases to protect mRNAs from degradation [54]. Nevertheless, in both cases, solid bodies protect *E. coli* mRNA from degradation during stresses. Once the stress subsides, BR‐ or HP‐bodies may disassemble, releasing stored mRNAs for translation [47].

Within HP condensates, Hfq may play an additional regulatory role by selectively stabilizing polyadenylated mRNAs [12]. Indeed, Hfq protects both the poly(A) tail and *rpsO* mRNA internal binding site from ribonuclease degradation (specifically from PNPase, RNase II, and RNase E [55]). Our results thus provide evidence for an additional RNA‐protecting mechanism, in which Hfq forms solid‐like bodies through its amyloid region, introducing a new layer of regulation for this important protein [12, 14]. This new finding reinforces the role of solid bodies as specialized storage compartments for mRNA to protect their integrity. Moreover, the use of an amyloid region reveals a new sophisticated strategy employed by bacteria to create cellular compartments without relying on lipid membranes. Note that the involvement of a functional amyloid region may contribute to the reversibility of HP‐bodies formation, enabling the release of stored transcripts when growth conditions change [19].

Note that polyP is also essential for Hfq's DNA‐binding behavior. Hfq and polyP form condensates with AT‐rich DNA, driven by Hfq's C‐terminal region (CTR) [40]. Our results, however, provide evidence that the Hfq:polyP:DNA complex does not induce Hfq amyloidogenicity *in vitro*.

Several questions remain unanswered. For example, what mechanism directs Hfq to HP‐bodies [39, 54]? How do HP‐bodies modulate noncoding sRNA‐mediated gene expression [56]? Another important question is where are these solid bodies located within the bacterial cell, especially since *E. coli* RNase E and Hfq are known to be membrane‐associated [20, 57, 58] (Fig. 6). However, the protein may be displaced from the membrane and diffuse into the cytoplasm under stress conditions [59]. Finally, while pole condensates and aggresomes have been shown to contain sRNAs [46, 54], BR‐bodies exclude structured RNA such as sRNA [48]. There is currently no evidence that HP‐bodies contain sRNA, a property that warrants further investigation, notably for stress responses where sRNA are key players [60] (Fig. 6).

In conclusion, PolyP seems to play a crucial regulatory role in modulating Hfq structure and function, particularly in the context of bacterial stress responses. Its involvement seems crucial for optimizing Hfq activity under challenging conditions by modulating protein interactions with polyadenylated RNAs. While additional work will be necessary to confirm that the suggested mechanism is active under physiological conditions, the underexplored C‐terminal region of the protein is likely to be a key factor in this process, underscoring its critical role in Hfq function [42, 49, 61, 62]. Additionally, PolyP might also modulate the membrane's physical properties and its interaction with HP‐bodies and Hfq [20]. Understanding the precise mechanisms by which PolyP interacts with both Hfq and its C‐terminal region may thus offer valuable insights into bacterial adaptation and stress resilience, with some implications for novel antibacterial strategies [56].

## Materials and methods

### Chemicals

All chemicals were purchased from Sigma‐Aldrich (Burlington, MA, USA) or Thermo fisher scientific (Waltham, MA, USA).

### Preparation of complexes for SRCD analysis

The sample analyzed consisted of the amyloid Hfq‐CTR peptide bound either to a double‐stranded (ds) DNA sequence: (dA : dT)_59_ or single‐stranded (ss) RNA. The choices of this homo‐polymeric DNA sequence and *rpsO‐polyA* were made because Hfq has highest affinity for A‐rich sequences [41, 42].

The C‐terminal region (CTR) of Hfq, corresponding to residues 64–102 of the full‐length protein, was chemically synthesized (ProteoGenix, Schiltigheim, France). The peptide sequence was as follows:

SRPVSHHSNNAGGGTSSNYHHGSSAQNTSAQQDS EETE,

and it was prepared at a concentration of 20 mg·mL^−1^ according to the protocol described by Fortas *et al*. [18].

Note that while only 11 amino acids of the C‐terminal region (CTR) are sufficient to drive Hfq amyloidogenesis (underlined in the CTR sequence), this short segment does not interact with nucleic acids [21] and therefore cannot be used for the present study.

RNA oligonucleotides were synthesized by Eurogentec (Seraing, Belgium). The sequences used were as follows:

For *rpsO* (corresponding to the 3′ end of *rpsO* mRNA):

CAGAAAAGGGGGCCUGAGUGGCCCCUUUUUUC.

*rpsO‐polyA* (polyadenylated version of *rpsO*):

CAGAAAAGGGGGCCUGAGUGGCCCCUUUUUUCAAAAAAAAAAAAAAAAAA.

For dsDNA, a (dA : dT)_59_, synthesized by Eurogentec (Belgium), was used.

All NAs were dissolved in sterile, molecular biology‐grade water at a final concentration of 1 mm (strand concentration) and relaxed for 2 min at 90 °C followed by a slow cool down before use.

Complexes between (dA : dT)_59_ and Hfq‐CTR peptides were prepared in water as described previously [24, 49]. Briefly, complexes were prepared at pH 5 and used at a final peptide concentration of 1.8 mm. While pH 5 may not be physiological and higher pH values should better approximate *in vivo* conditions, the microenvironment within a densely packed protein or complex can deviate significantly from cytoplasmic pH 6.8–7.0, and the pH in granules may also differ [63]. Initially, we examined CTR–nucleic acid complex formation at pH 7.5 but observed substantially reduced binding affinity under these conditions, the reason we shifted the pH to 5 [21, 64]. It is also important to note that *in vivo*, the CTR is not isolated but associated with the N‐terminal region of Hfq, which may modulate the local microenvironment of CTR residues, potentially altering their pKa values and net charge at physiological pH. Note that at the concentrations used, the peptide exhibits self‐buffering properties.

Salts were omitted from SRCD analyses, as commercially sourced DNA and peptides are resuspended in water and contain residual salts sufficient for these experiments. As additional salts do not substantially change the affinity of the CTR for DNA [21], we preferred to omit them in our preparations in order to get a broader spectral band (170–320 nm) [34, 65]. This approach follows the protocol used in the accumulation of the SP175 reference dataset (https://pcddb.cryst.bbk.ac.uk/) [66].

The stoichiometry was 1 Hfq‐CTR per 1 nucleotide for *rpsO* RNA, per 4 base pairs for DNA and per 1 phosphate for polyP. The ratio CTR/NA was increased with DNA due to a lower affinity compared with RNA [41].

Samples were analyzed after at least 4 weeks to allow peptide self‐assembly on DNA, a process that is not instantaneous [24].

### Synchrotron radiation circular dichroism (SRCD)

SRCD measurements were performed at the DISCO beamline at SOLEIL Synchrotron, following the procedure outlined by Malabirade *et al*. [24] (proposal 20241127). Approximately 4 μL of sample was placed into a 33 μm pathlength CaF₂ circular cell. Spectra were acquired in triplicate at 1 nm intervals with an integration time of 1.2 s, covering the range from 180 to 320 nm. Calibration of amplitude and wavelength positions was carried out using (+)‐camphor‐10‐sulfonic acid (CSA). Data analysis, including averaging, baseline subtraction, smoothing, scaling, and spectral summation, was performed using cdtoolx [67]. The resulting spectra are presented in millidegrees (mdeg) versus nanometers (nm), with the same molar ratios maintained across all samples. Due to the nature of the absorption, spectra of mixed samples (polynucleotides + peptides) could not be standardized to ∆*ε*. CTR secondary structure content was determined using BestSel [68].

### Negative stain transmission electron microscopy

A 3.5 μL aliquot of sample was applied to glow‐discharged Formvar–carbon–coated copper grids (200‐mesh; Electron Microscopy Sciences, Hatfield, PA, USA). Grids were glow‐discharged for 60 s at 25 mA using a GloQube Plus system (MiTeGen, Ithaca, NY, USA). After a 60 s adsorption period, excess liquid was removed with Whatman Grade 1 filter paper. Grids were rinsed three times with 20 μL Milli‐Q water and stained with 2% (w/v) uranyl acetate (Electron Microscopy Sciences). Stained grids were air‐dried for 30 min at room temperature and subsequently examined using a 120 kV FEI TALOS transmission electron microscope equipped with a single‐tilt, room temperature holder. Images were acquired at nominal magnifications of ×28 000 and ×45 000. Micrographs were recorded and processed using velox software.

### Attenuated Total reflection Fourier transform infrared spectroscopy (ATR‐FTIR) analysis

ATR‐FTIR measurements were performed as described previously in Turbant *et al* [32].

## Author contributions

Conceptualization, VA; methodology, KM, SB, FW, FT, and VA; software, KM, SB, and FT; formal analysis, KM, SB, and VA; investigation KM, SB FW, and VA; data curation, KM and SB; writing original draft preparation, KM, SB, RS, and VA.; writing – review and editing, KM, SB, FT, FW, RS, and VA.; visualization, KM and VA.; project administration, VA.; funding acquisition, VA; All authors have read and agreed to the published version of the manuscript.

## Conflicts of interest

The authors declare no conflicts of interest.

## Data Availability

SRCD, FTIR, and TEM data are available from http://doi.org/10.5281/zenodo.18312686.
